# Incidence of Using Physical, Mechanical Restraints and Seclusion in Saudi Mental Health Settings: A Prospective Cohort Study

**DOI:** 10.3390/healthcare14081011

**Published:** 2026-04-12

**Authors:** Asrar Salem Almutairi, Antonia Marsden, Owen Price, Abdullah Hassan Alqahtani, Abdullelah Waleed Almulhim, Saleh Alsaidan, Modhi Alanazi, Karina Lovell

**Affiliations:** 1 Division of Nursing, Midwifery and Social Work, University of Manchester, Manchester M13 9PL, UK; owen.price@manchester.ac.uk (O.P.); karina.lovell@manchester.ac.uk (K.L.); 2 College of Nursing, Princess Nourah Bint Abdulrahman University, Riyadh 84428, Saudi Arabia; 3 Division of Population Health, Health Services Research & Primary Care, University of Manchester, Manchester M13 9PL, UK; antonia.marsden@manchester.ac.uk; 4 Psychiatry Division, Medicine Department, Johns Hopkins Aramco Healthcare (JHAH), Dhahran 31311, Saudi Arabia; dr.alqahtani16@gmail.com; 5 Eradah Complex and Mental Health-Dammam, Dammam 32312, Saudi Arabia; elahmulhim@gmail.com; 6 Department of Psychiatry, College of Medicine, King Fahad University Hospital, Imam Abdulrahman Bin Faisal University, Dammam 34212, Saudi Arabia; smaalsaidan@iau.edu.sa; 7 Eradah Complex and Mental Health-Qurayyat, Qurayyat 75911, Saudi Arabia; moaalanazi1@moh.gov.sa

**Keywords:** incidence, physical restraints, mechanical restraints, seclusion, mental health, Saudi Arabia

## Abstract

Background/Objectives: The use of physical and mechanical restraints and seclusion in psychiatric facilities to manage violent and aggressive behaviours has long been a contentious issue, balancing patient safety with ethical considerations. With advancements in psychiatry and increased understanding of mental illness, there have been expectations that such interventions would no longer be required; however, their use persists in clinical practice. Management policies differ across countries and are largely influenced by legal frameworks. This study aimed to identify the factors influencing the incidence of these interventions across two psychiatric facilities in Saudi Arabia and to examine associations among inpatient variables. Methods: A prospective cohort study was conducted over six months (September 2021–March 2022) across two psychiatric facilities in Saudi Arabia (Eradah Complex, *n* = 1120; King Fahd University Hospital (KFUH), *n* = 268). Data from 333 restriction events were analysed using descriptive statistics, chi-square tests, and negative binomial regression to calculate incidence rates and explore associated factors. Results: The findings revealed a complex interplay of factors related to patient characteristics and clinical and environmental conditions within the facilities. Key contributing variables included symptom deterioration and the duration of observation required. Longer observation periods were associated with certain diagnoses, particularly schizophrenia and mood disorders. Conclusions: Restraints and seclusion remain influenced by multiple interacting factors within psychiatric settings. These findings highlight the need to reduce their use and ensure they are applied cautiously, with greater emphasis on minimising patient trauma and promoting safer, person-centred care.

## 1. Introduction

### 1.1. Background

The rates of use and patterns of restraint and seclusion in mental health settings highlight several important aspects regarding treatment and care practices in this context [1]. Studies show that there are significant differences in the deployment of restraint and seclusion between various countries and cultures, indicating major challenges facing policies and practices in this area [2,3]. One study suggests that some countries, such as Iceland, prefer to avoid the use of restrictions and isolation altogether, while others, such as Austria, see high usage rates for these measures. This reflects differences in medical culture and health policies between countries, as well as the influences of social, economic, and cultural factors on health care practices [4]. Rates and patterns of restraint and seclusion are influenced by a complex interplay of factors. These can be broadly categorised into patient, staff, and institutional determinants. Regarding patient factors, variables such as age, gender, psychiatric diagnosis (e.g., schizophrenia, mood disorders), and the reason for admission (e.g., risk of aggression) have been shown to affect the likelihood of experiencing these interventions [5,6,7]

Despite the extensive international literature on restraint and seclusion, several critical knowledge gaps exist within the Saudi Arabian context. First, no empirical data are available on the baseline incidence of these coercive practices in Saudi mental health settings, making it impossible to assess whether local practice aligns with international standards or to monitor the impact of future policy changes. Second, while international studies have identified patient-level, staff-level, and institutional determinants of restraint and seclusion use, it remains unknown whether these factors operate similarly in the Saudi context, where cultural norms, family involvement, and healthcare delivery models differ substantially from Western settings where most research has been conducted. Third, no studies have compared practices across the two main types of psychiatric facilities in Saudi Arabia, specialised government psychiatric hospitals and psychiatric units within general teaching hospitals, to understand how setting characteristics influence practice patterns. Fourth, the relationship between nursing workforce characteristics (ratios, education, experience) and the use of coercive measures has never been examined in Saudi Arabia, despite evidence from other countries that these factors are critical determinants [8,9].

This study addresses these gaps directly. By prospectively collecting data from two distinct facilities, we provide the first-ever baseline incidence rates for restraint and seclusion in Saudi Arabia. By analysing patient demographic and clinical characteristics, we identify which patient populations are most at risk for experiencing coercive interventions in this context. By comparing practices across hospital types and settings, we illuminate how institutional factors shape decision-making. And by collecting daily nursing data, we examine the relationship between staffing characteristics and the use of restrictive measures. Together, these contributions establish an evidence base that can inform culturally appropriate policies and interventions to reduce coercive practices in Saudi mental health settings.

Donat examines the impact of improved staffing on the reliance on seclusion and restraint in a public psychiatric hospital. The results suggest that better staffing levels contribute to a decreased need for seclusion and restraint practices. The study highlights how enhanced staff–patient interactions and increased staff availability can lead to safer and more therapeutic environments, ultimately reducing negative effects such as anger, fear, and distress among patients, as well as decreasing the risk of violence and injury [8]. In terms of staff factors, characteristics like education, experience, and the number of staff available play a significant role [5,6]. For example, Donat [8] found that improved staffing levels contributed to a decreased need for seclusion and restraint. Another study focuses on patients’ reports of traumatic or harmful experiences within psychiatric settings. The findings indicate that practices like seclusion and restraint can have profoundly negative impacts on patients, including inducing trauma and exacerbating fear and distress. The study calls for the consideration of patients’ experiences in the development of policies and emphasises the need for alternatives that prioritise patient safety and well-being [10]. A key study explores patients’ experiences with seclusion and restraint in psychiatric care and provides practical suggestions for improving practices and utilising alternatives. The patients’ narratives reveal feelings of anger, fear, and helplessness, underscoring the negative psychological impact of such interventions. The study suggests that involving patients in treatment decisions and focusing on therapeutic approaches can significantly enhance the care environment and reduce the need for restrictive practices [9].

Conversely, other research suggests that the relationship is not linear, with Staggs et al. [11] arguing that increased staffing could, in some contexts, heighten the rate of violence. Therefore, the staffing ratio can be an influential factor and should be taken into consideration in mental health care settings. The seclusion-related incident rate represents the number of cases in which seclusion was used in 100 cases, or per specified number of population or cases during a given period of time [12]. The rate of isolation-related incidents is affected by several factors, including patient characteristics such as age, gender, nationality, and psychiatric diagnosis. Violence experienced by healthcare workers could be a major reason for the use of seclusion, a study has found [13].

Finally, institutional and environmental factors, such as the time of day, ward atmosphere, training programmes, and organisational policies, are also critical in determining the use of these coercive measures [7]. They used a comprehensive analysis to evaluate the patient-related, environmental, and structural factors of the clinic, and found that aspects such as age, gender, history of violence and certain psychiatric disorders could be associated with increased coercive measures [7]. Environmental conditions within the hospital play an important role in determining the rates of isolation use, such as increased pressure on nurses at certain times, a sufficient number of health care workers, training programmes and organisational policies followed in health institutions.

The practice of using restraint and seclusion in psychiatric hospitals is a critical issue in mental health care, particularly concerning the safety, care, and rights of patients. To ensure conceptual clarity, in this study, we define these interventions as follows: Physical restraint refers to a hands-on method by staff to restrict a patient’s movement. Mechanical restraint involves the use of devices (e.g., belts or straps) attached to the patient’s body to limit their movement, which cannot be easily removed by the patient. Seclusion is defined as the involuntary confinement of a patient alone in a room or area from which they are physically prevented from leaving.

Recent systematic reviews have synthesised evidence on effective interventions to reduce coercive practices, highlighting the importance of organisational culture change, staff training, and post-incident review; see Baker et al., 2021 [14].

Notably, the use of restraint and seclusion varies significantly across different countries, influenced by varying legal frameworks, cultural attitudes, and clinical guidelines. This disparity highlights the importance of examining these practices within specific national contexts to understand better and address their unique challenges and impacts.

While international literature documents the use of restraint and seclusion, there is a significant lack of empirical data on their incidence and the factors influencing their use within the mental health system of Saudi Arabia. The cultural context, healthcare policies, and the structure of psychiatric services in the Kingdom are distinct. For instance, mental health services are provided by both large government psychiatric hospitals and smaller units within general teaching hospitals, yet no study has systematically compared practices across these settings. This study aims to fill this knowledge gap by providing the first prospective data on the incidence and patterns of these interventions in two major Saudi facilities, thereby establishing a baseline to inform culturally sensitive policy development and restraint reduction programmes.

In summary, while the use of coercive measures is a global challenge, the specific determinants within the Saudi healthcare system remain unexplored. Therefore, this study aims to address this gap by examining the incidence and patterns of physical/mechanical restraints and seclusion in two Saudi mental health settings. The specific objectives were to

- Descriptive Objectives

1. Summarise demographic and clinical information of patients in the two settings.
2. Summarise information about restraint and seclusion events, including duration, timing, type, and reasons for use.
3. Summarise nursing characteristics, including nurse-to-patient ratios, educational levels, and years of experience.

- Analytical Objectives

1. Explore associations between patient factors (age, gender, diagnosis) and reason for admission.
2. Explore associations between patient factors and duration of observation.
3. Explore associations between hospital type, setting, and the type and duration of restrictive interventions.
4. Explore associations between nursing factors (ratios, education, experience) and the incidence of restraints and seclusions.

### 1.2. Significance

Understanding the rates and patterns of use of restraint and seclusion in mental health settings in Saudi Arabia is of great importance. By conducting such research, we can uncover the reasons behind the use of these interventions and identify influencing factors. This understanding is critical to developing and implementing successful restraint reduction programmes in the future. By addressing the causes and root factors that contribute to the use of restraint and seclusion, adapted strategies can be developed to reduce their use in mental health facilities across Saudi Arabia. Ultimately, this improves patient care, enhances patient rights, and promotes a safer and more therapeutic environment for individuals receiving mental health treatment in Saudi Arabia.

## 2. Materials and Methods

### 2.1. Study Design

This study is a prospective, observational cohort study and was designed to observe the incidence of physical/mechanical restraints and seclusion on patients during a six-month period. Alongside this was the intention to investigate the relationship between demographic, clinical and hospital characteristics and the use of physical/mechanical restraints and seclusion among adult psychiatric inpatients. The study was conducted after obtaining approval from the appropriate Institutional Ethics Committee and in accordance with the Declaration of Helsinki, good clinical practice, and local ethical and legal requirements.

### 2.2. Data Sources

The study was conducted at two key healthcare facilities in the eastern region of Saudi Arabia: Eradah Complex and Mental Health in Dammam, and King Fahad University Hospital in Dammam. Eradah Complex and Mental Health, a government psychiatric hospital, has a substantial capacity of 500 beds and serves as a primary mental health care provider in the region. King Fahad University Hospital, with its 36-bed psychiatric unit, is integral for training and research, offering a contrast in setting and scale. This selection allows for a comparative analysis of restraint and seclusion practices across different hospital types and capacities, reflecting a broader spectrum of psychiatric care environments. Data collection spanned six months, from 5 September 2021 to 5 March 2022. During this period, all patients admitted to the participating wards were enrolled in the study cohort, and data were systematically gathered from two primary sources: (1) clinical records of patients and (2) daily nursing shift reports.

Regarding patient data, approved psychiatrists at each facility used a standardised quantitative data collection form to extract information from electronic and paper clinical records. This form captured detailed information for each admitted patient, including demographic characteristics; psychiatric diagnoses (coded according to ICD-10); reasons for admission; hospital and departmental settings; admission and discharge dates (to calculate duration of observation); and, where applicable, the timing, duration, type, and reasons for any restraint or seclusion events. All data were anonymised at the point of extraction by removing patient identifiers (name, national ID, medical record number) before being transferred to the research team.

To ensure data quality and reliability, the following measures were implemented. First, all psychiatrists involved in data extraction received standardised training on the data collection form and coding procedures. Second, a random sample of 10% of records was independently re-extracted by a second data collector at each site, and inter-rater reliability was assessed, yielding a kappa coefficient of >0.85 across all variables, indicating excellent agreement. Third, data were checked for completeness and consistency on a weekly basis by the lead researcher, with queries resolved through consultation with the site data collectors. Fourth, where discrepancies or missing data were identified, original records were re-accessed (where permitted by ethical approvals) to verify and complete the information.

Regarding nursing data, detailed information on staff characteristics was collected daily from shift reports. This included total numbers of nurses on duty per shift, nurse-to-patient ratios, educational levels (diploma, bachelor’s, postgraduate), and years of experience in psychiatric settings (categorised as <1 year, 1–5 years, 6–10 years, >10 years). These data were recorded separately for each ward and each shift to capture day-to-day variations in staffing.

### 2.3. Study Population

This study encompassed a dynamic cohort comprising all patients admitted to nine participating wards across the two facilities during the six-month study period (5 September 2021, to 5 March 2022). The cohort included both emergency and inpatient admissions.

Inclusion criteria: All patients aged 18 years and older who were admitted to any of the participating wards during the study period were included in the cohort and followed for the duration of their admission.

Exclusion criteria: The only exclusion criterion was age under 18 years. This age restriction was applied because the study focused on adult psychiatric populations, and paediatric mental health services operate under different regulatory frameworks and clinical protocols in Saudi Arabia. No other exclusion criteria were applied, ensuring that the cohort represented the complete population of adult psychiatric inpatients at these facilities during the study period.

The total cohort comprised 1388 unique patients: 1120 patients admitted to Eradah Complex and Mental Health and 268 patients admitted to King Fahad University Hospital. For each patient, the observation period began at admission and ended at discharge. Patients readmitted during the study period were treated as new admissions, with separate observation periods, consistent with the dynamic cohort design. The total observation days across all patients were calculated and used as the offset variable in the incidence rate models, as described in Section 2.4.

### 2.4. Statistical Analysis

In this study, all incidents involving physical/mechanical restraints or seclusion were analysed to determine the incidence of these events. Descriptive statistics were utilised to summarise key variables, such as the time of day, type of hospital, setting, reasons for restrictions, and duration of restriction. Data were categorised by the type of restriction and presented in terms of percentages and absolute frequencies. Similarly, patient characteristics (including diagnosis and reason for admission), along with nurse characteristics (such as nurse-to-patient ratio, educational level, and experience), were analysed based on the type of hospital where the events occurred.

To examine the relationships between the type of restrictions and categorical factors such as time of day, hospital type, setting, and reasons for restrictions, the chi-square test was employed. Additionally, the Kruskal–Wallis test was used to analyse the variable ‘Age’ in relation to the reason for admission and the duration of observation.

A negative binomial model was used to calculate the incidence rate of seclusion and of physical/mechanical restraint. The associations between patient and nurse characteristics and the incidence of events were also explored using this model, with results reported as **Incidence Rate Ratios (IRRs)**. A negative binomial model was used to calculate the incidence of seclusion and that of physical/mechanical restraint. This model was chosen over the Poisson model because the data was over-dispersed, i.e., the variance of the outcome was greater than the mean [15]. Six separate models were fitted to estimate six separate incidence rates: (1) incidence of physical/mechanical restraint in Eradah; (2) incidence of seclusion in Eradah; (3) combined incidence of physical/mechanical restraint and seclusion in Eradah; (4) incidence of physical/mechanical restraint in KFUH; (5) incidence of seclusion in KFUH; and (6) combined incidence of physical/mechanical restraint and seclusion in KFUH. The outcomes were the number of mechanical restraints, number of seclusions, and the total number of both in three different models. No covariates were included at the outset to allow the estimation of the overall incidence. Duration of observation per patient was used as an offset variable to account for the fact that the length of stay was different for each patient. Incidence was calculated as a number of events per month. The associations between patient and nurse characteristics and the incidence of events were also explored using this model by including these as covariates in the model. To explore associations between nursing factors and the incidence of restraints and seclusions, we fitted separate negative binomial models for each hospital. Based on the previous literature [5,6,7,8], we hypothesised that the total number of nurses on shift, the number of nurses with diploma-level education, and the number of nurses with 1–5 years of experience would be key predictors and were therefore included as covariates in the models.

All statistical analyses were performed using IBM SPSS Statistics software, version 25.0. (IBM Corp., Armonk, NY, USA).

## 3. Results

### 3.1. Descriptive Analysis

#### 3.1.1. Patient Data

Table 1 provides a detailed summary of patient demographics and clinical characteristics at the two hospital settings, the psychiatric hospital (Eradah) and the teaching hospital (KFUH), comprising a total of 1388 patients.

Firstly, regarding age distribution, both hospitals primarily serve a younger demographic, with the majority of patients aged between 20 and 39 years, representing approximately 63% of the total population across both institutions. The average age of patients at Eradah is slightly higher at 35.42 years compared to 34.58 years at KFUH, with the overall mean age being 35.26 years.

In terms of gender, there is a notable predominance of male patients in both hospitals, with males making up 67.3% of the psychiatric patient population in Eradah and 55.6% in KFUH. Overall, males account for 65.1% of the combined patient cohort, suggesting a significant gender disparity in psychiatric admissions.

Diagnostically, schizophrenia and other non-mood psychotic disorders (F20–F29) dominate at Eradah, affecting 69.7% of its patients, while mood disorders (F30–F39) are more prevalent at KFUH, impacting 56.7% of its patients. This difference underscores the specialised focus of Eradah on severe psychiatric conditions and the broader, perhaps more educational focus at KFUH.

The primary reasons for admission across both facilities include worsening of symptoms (39.9%), risk of aggression (30.5%), and risk of suicide (22.3%). The low percentages for diagnostic uncertainty and risk of moral exposure suggest that most admissions are driven by clear clinical presentations and immediate risks.

Lastly, the setting type data reveals a high proportion of Emergency Room (ER) admissions, constituting 43.9% of all cases, indicative of the acute nature of many psychiatric admissions. There is also a greater number of male ward admissions (38.3%) compared to female wards (17.9%), further highlighting the gender disparity in these healthcare settings.

#### 3.1.2. Restriction Data

Table 2 focuses on the use of restrictive interventions—specifically mechanical or physical restraint and seclusion. It provides insights into the duration and timing of these interventions, the types of hospitals and settings in which they are employed, and associated behaviours such as violence or damage.

##### Duration and Timing of Restrictions

A total of 333 instances of restriction were reported, with mechanical or physical restraint being more commonly used (238 instances) compared to seclusion (95 instances). The majority of restrictions lasted between 0 and 60 min, with 83.4% of total instances falling within this time frame, highlighting that shorter durations are preferred or sufficient in most cases. The breakdown indicates a slightly higher percentage of longer durations (over 121 min) in mechanical restraint scenarios than in seclusion, suggesting that more severe or challenging cases might be managed with physical restraints.

Restrictions are fairly evenly distributed throughout the day, with a slight decrease during the night (18.9% of instances). This might indicate that daytime activities and interactions could potentially increase the need for restrictions, or that nighttime staffing levels or patient activities decrease the incidence or reporting of such interventions.

##### Types of Hospital and Setting

A significant majority of the restrictions occur in psychiatric hospitals (82.6%), which is expected given the nature of patient needs and the clinical focus on managing psychiatric symptoms that might lead to self-harm or aggression. The teaching hospitals, while having a lower overall percentage, still utilise these interventions, indicating their role in managing acute psychiatric situations or in training settings.

Regarding the settings within hospitals, there is a notable difference in the application of restraints or seclusion between male and female wards. Mechanical restraint is predominantly used in male wards (79% of instances in male settings), reflecting perhaps higher occurrences of aggression or other behaviours requiring such interventions. In contrast, seclusion is more commonly used in female wards (76.8% of seclusion instances), which may suggest different strategies for managing crises or behavioural episodes between genders.

##### Departmental and Behavioural Contexts

Within the psychiatric departments, there is a higher utilisation of mechanical restraint in departments with male patients, while female wards show a significant reliance on seclusion. This could reflect differing approaches based on patient demographics or symptomatology.

Regarding behaviours associated with the use of restraints and seclusion, there is a substantial report of violence towards self, staff, and other patients, as well as damage to the environment as precursors to deploying R/S. About a quarter of the cases involved self-violence and violence towards patients, while a slightly higher percentage (33.3%) involved violence towards staff. These figures underscore the challenging environments in which clinical staff operate and the potential impetus behind this level of restraint use.

#### 3.1.3. Daily Data and NURSING Characteristics

Daily data refers to the collection of information based on the nursing staff present and working during each day of the study period. This approach provides dynamic and accurate insights into staffing patterns, reflecting variations in nurse-to-patient ratios, educational qualifications, and experience levels on a day-to-day basis rather than providing a statical overview of the total nursing workforce employed by each hospital.

Table 3 represents the nursing characteristics at Eradah and KFUH. Starting with the nurse-to-patient ratios, Eradah has a median of 0.25 (ranging from 0.19 to 0.32), indicating that each nurse typically covers four patients. In contrast, KFUH has a much higher median ratio of 1.1 (ranging from 0.67 to 1.89), where each nurse manages slightly less than one patient on average.

Regarding the educational background of nurses, Eradah employs a significantly higher number of diploma-level nurses, with a median daily number of 28 (ranging from 19 to 40), compared to a daily median of just 5 at KFUH (ranging from 3 to 6). Conversely, KFUH has a higher median daily number of bachelor-prepared nurses, 12 (ranging from 7 to 17), compared to 9 at Eradah (ranging from 2 to 15). These figures represent the average daily number of nurses on shift during the study period, reflecting staffing patterns per day rather than the overall number of nurses employed at each hospital.

In terms of experience, both hospitals exhibit diverse experience levels, but Eradah Hospital generally employs more nurses across different experience categories, particularly in the mid- to high-experience ranges. For example, Eradah has 14 nurses with 6 to 10 years of experience and 11 with more than 10 years, compared to KFUH’s 6 and 9, respectively. This suggests that Eradah might have a more experienced workforce overall.

### 3.2. Statistical Analysis

#### 3.2.1. Restrictions Data

Table 4 represents the association between the type of restrictions, the type of hospital, the type of setting, and the duration of restrictions.

Regarding the manner of restrictions, a distinct pattern in the duration of restrictions was observed based on their type. For mechanical or physical restrictions, a majority (74.2%) lasted 0–30 min, followed by 25.8% lasting 31–60 min. However, seclusion shows a varied pattern, where 63.3% of restrictions lasted 0–30 min, and a notably higher proportion (36.7%) lasted 31–60 min compared to mechanical restrictions. More strikingly, long durations (61+ minutes) are significantly more common in seclusion (10.9%) than in mechanical restrictions (0%). This suggests that seclusion tends to be used for longer periods compared to mechanical or physical restraints. The statistical significance of these differences is confirmed by a *p*-value of <0.001, indicating a strong association between the type of restriction and the duration for which it is applied.

In terms of the type of hospital, the restriction duration also varies notably between different types of hospitals. In psychiatric hospitals, a predominant majority of restrictions (82.5%) last 0–30 min, with only 17.5% extending to 31–60 min. In contrast, teaching hospitals have a higher frequency of longer restrictions, with 89.9% lasting 0–30 min and 10.1% extending to 31–60 min. Furthermore, longer durations (61+ minutes) are more prevalent in teaching hospitals (38.2%) compared to psychiatric hospitals (18.2%), indicating a possible trend towards longer durations of restrictions in teaching settings, which might reflect differences in patient demographics, case complexities, or institutional policies. The *p*-value of <0.001 highlights the statistical significance of these findings, underscoring the important differences in how restrictions are managed in psychiatric versus teaching hospitals.

Regarding the type of setting, within the hospital locale, the male ward, female ward, and emergency room (ER) show different patterns of restriction durations. The male ward has 64.2% of restrictions lasting 0–30 min and 30.8% for 31–60 min. The female ward shows a greater lean towards shorter durations, with 55.1% of restrictions lasting 0–30 min and 43.7% for 31–60 min. Notably, the ER has the highest proportion of longer durations, with 81.8% of restrictions lasting 0–30 min but only 18.2% for 31–60 min. The significant differences across these settings are statistically validated with a *p*-value of <0.001, indicating a substantial impact of the hospital setting on the duration of restrictions.

Table 5 shows the association between the type of restriction and the type of setting. Physical or mechanical restraints are predominantly used in the male ward, with 188 instances accounting for 79% of such interventions. Physical or mechanical restraints were used in 188 instances in the male ward, accounting for 79% of such interventions. Table 5 presents the association between the type of restriction and the type of setting. Physical or mechanical restraints were predominantly used in the male ward, with 188 instances accounting for 79.0% of such interventions. In contrast, seclusion was significantly more prevalent in the female ward, used in 73 cases, which represents 76.8% of the total seclusion instances. Seclusion was used much less frequently in the male ward (22.1%) and was very rare in the emergency room (1.1%). The association between the type of setting and the type of restraint used was statistically significant (*p* < 0.001, Fisher’s Exact Test).

#### 3.2.2. Association Between Patient Characteristics and Their Reasons for Admission at a Hospital

Table 6 represents the association between patient characteristics and their reasons for admission at a hospital (N = 1231) and provides a comprehensive view, with statistically significant differences across various parameters including age, gender, diagnosis, setting, and the type of hospital (*p* < 0.001).

##### Age and Reason for Admission

Patients admitted due to the risk of suicide had a mean age of 33.46 years; those admitted for risk of aggression averaged 34.96 years; and those with worsening symptoms were typically older, with a mean age of 36.49 years. These mean ages are statistically significantly different, with a *p*-value of <0.001. This suggests that older patients tend to be admitted more for worsening symptoms, whereas younger patients are more frequently admitted for reasons related to immediate risks such as suicide and aggression.

##### Gender Distribution

Gender distribution also showed significant differences (*p*-value < 0.001) across reasons for admission. Males constituted 56.1% of admissions for suicide risk, 77.8% for aggression risk, and 63.5% for worsening symptoms, indicating a higher proportion of males being admitted for aggression. Females comprised 43.9%, 22.2%, and 36.5% of admissions for these reasons respectively, which underscores a gender disparity particularly notable in admissions for aggression.

##### Diagnosis

Diagnosis upon admission revealed significant variations (*p*-value < 0.001) among the patient groups. For instance, 44.6% of those admitted for risk of suicide were diagnosed with schizophrenia, schizotypal delusions, or other non-mood psychotic disorders, while this figure rose dramatically to 70.6% and 69.9% for those admitted for aggression and worsening symptoms, respectively. In contrast, mood disorders were more commonly associated with suicide risk admissions (41.8%) compared to aggression (21.8%) and worsening symptoms (25.2%).

##### Hospital Setting and Type

Hospital setting also showed a significant association with the reason for admission (*p*-value < 0.001). The ER accounted for 46.5% of suicide risk admissions, 37.3% for aggression, and 45.3% for worsening symptoms, suggesting that the ER is a common point of entry for urgent psychiatric care. The differences in settings might reflect the urgency and type of response required by the patient’s condition at admission.

#### 3.2.3. Association Between Patient Characteristics and Duration of Observation at a Hospital

The results presented in Table 7 show that age is not significantly associated with duration of observation (*p* = 0.068). Similarly, there was no significant association between gender and duration of observation (*p* = 0.16). Furthermore, the results show that the observed variations in the prevalence of disorders across different time periods are statistically significant (*p* < 0.008). Mental and behavioural disorders due to psychoactive substances decreased significantly with longer observation periods. Schizophrenia and other non-mood psychotic disorders exhibited an increasing trend over time. Mood disorders showed a mixed pattern, with a decrease initially and a slight increase later. Anxiety and other nonpsychotic disorders remained relatively stable across observation periods.

The results indicate a statistically significant association between the setting and the duration of observation. The male ward exhibited a higher proportion of patients with shorter observation periods. Conversely, the female ward showed a more even distribution across the observation durations. Notably, the emergency room (ER) had a dominant presence of patients with observation periods less than 30 days, and no patients with durations between 30 and 90 days. The results show that there was no significant association between Hospital setting and duration of observation (*p* = 0.098). The median age for patients admitted due to risk of suicide was 31 years (IQR: 24–41), compared to a median of 34 years (IQR: 27–43) for those with worsening symptoms Lastly, the results reveal a significant difference in admission reasons across different observation periods (*p* < 0.001). Shorter observation periods are linked to a higher percentage of admissions due to the risk of suicide and aggression, while longer periods show a greater prevalence of admissions related to the worsening of symptoms.

#### 3.2.4. Daily Data

##### Incidence of Physical/Mechanical Restraints, Incidence of Seclusions in Eradah Complex and Mental Health and KFUH

The model outputs provide the average number of events per person per day. To make the results more interpretable, the values were multiplied by 30.5, reflecting the average number of days in a month. This adjustment allows the data to be presented as the average number of events per person per month, offering a more intuitive understanding of the incidence rates (Table 8).

Table 8 presents the results of the negative binomial regression models estimating the incidence of restrictive interventions at each hospital.

- Eradah Complex and Mental Health

The model estimated a daily incidence rate of 0.007 mechanical restraint events per patient (Exp(B) = 0.007), which corresponds to a monthly rate of 0.2135 events per patient. For seclusions, the daily rate was 0.003 events per patient, equivalent to 0.0915 events per patient per month. The combined rate for any restrictive intervention was 0.010 events per patient per day, or 0.3050 events per patient per month.

- King Fahad University Hospital (KFUH)

At KFUH, the daily incidence rate for mechanical restraints was 0.012 events per patient, yielding a monthly rate of 0.3660 events per patient. The daily rate for seclusions was 0.005 events per patient, corresponding to 0.1525 events per patient per month. The combined rate for any restrictive intervention was 0.018 events per patient per day, or 0.5490 events per patient per month.

These findings indicate that the overall incidence of restrictive interventions was higher at KFUH compared to Eradah Complex, with monthly rates of 0.5490 versus 0.3050 events per patient, respectively.

##### Patients’ Factors Associated with the Incidence of Physical/Mechanical Restraints and Incidence of Seclusions in the Eradah Complex and Mental Health and KFUH

Table 9 presents the negative binomial regression analysis examining patient-related predictors of the incidence of physical/mechanical restraints and seclusion. It is important to note that several estimates, particularly the extremely large coefficients for male gender (B = 25.961; IRR = 1.8 × 10^10^) and female ward setting (B = 25.755), suggest potential model instability. These extreme values likely indicate problems such as quasi-separation (where one predictor perfectly or nearly perfectly predicts the outcome), sparse data in certain subgroups, or possible coding issues. Additionally, the use of a small reference category for diagnosis (‘Disorders of adult personality and behaviour,’ representing only 3.1% of the sample) may have contributed to unstable estimates. Therefore, these findings should be interpreted with appropriate caution, and the extreme values should be viewed as indicative of strong associations rather than precise effect sizes.

With these caveats in mind, the model suggests several patterns. Male gender showed an extremely strong association with increased incidence of restrictive interventions, though the precise magnitude cannot be reliably estimated from these data. Compared to patients with personality disorders, those with substance-related disorders (IRR = 10.83), mood disorders (IRR = 5.73), and schizophrenia spectrum disorders (IRR = 4.92) all showed substantially higher incidence rates. Patients admitted due to risk of aggression had significantly higher incidence compared to those admitted for worsening of symptoms (IRR = 1.60; *p* = 0.008). Increasing age was associated with a small but significant reduction in incidence (IRR = 0.96 per year; *p* < 0.001). Patients at Eradah Hospital had significantly lower incidence compared to those at KFUH (IRR = 0.64; *p* = 0.032).

Regarding the reason for admission, risk of aggression has a coefficient of 0.468 with a standard error of 0.1775, resulting in a Wald Chi-Square of 6.968, which is significant (*p*-value = 0.008). The Exp(B) value of 1.598 indicates that patients admitted due to a risk of aggression are about 1.6 times more likely to experience restraints or seclusions compared to those admitted for worsening of symptoms.

In addition, for age as a predictor, each further year of age is associated with a slight decrease in the likelihood of experiencing restraints or seclusions, with a coefficient of −0.043 and a highly significant Wald Chi-Square of 29.897 (*p*-value = 0.00). The Exp(B) value of 0.958 suggests that with each additional year, the likelihood decreases by about 4.2%.

Finally, regarding to the hospital as a predictor, being in Eradah Complex and Mental Health is associated with a lower incidence of restraints or seclusions compared to KFUH, with a coefficient of −0.444, standard error of 0.207, and a Wald Chi-Square of 4.589, which is significant (*p*-value = 0.032). The Exp(B) value of 0.641 indicates a 36% lower likelihood at Eradah Hospital.

##### Incidence of Physical/Mechanical Restraints and Seclusion and the Association with Nurses’ Factors for Eradah Complex and Mental Health

Table 10 explores the associations between the occurrence of physical/mechanical restraints and seclusion at the Eradah Complex and Mental Health hospital. We employed Negative Binomial Regression, specifically focusing on the factors of educational level among nurses with diplomas only, and nurses with only 1 to 5 years of experience.

Regarding the total number of nurses in Hospital Eradah, the incidence rate ratio (Exp.B = 0.621) indicates that an increase in the total number of nurses is associated with a decrease in the incidence of restraints and seclusions. The Wald Chi-Square value of approximately 27.80, *p* < 0.001 indicates a statistically significant relationship between the number of nurses and these incidents.

Regarding the total number of nurses with diploma-level education, the incidence rate ratio (Exp.B = 1.352) implies that as the number of diploma-level nurses increases, the incidence of restraints and seclusions also increases. This could suggest that while increasing the overall number of nurses reduces the frequency of restraints and seclusions, the level of education among the nurses may also influence the effectiveness of this reduction. The Wald Chi-Square value of about 11.25 (*p* = 0.001) suggests a statistically significant association between this variable and occurrences of physical/mechanical restraint and seclusion.

Furthermore, for the total number of nurses with 1 to 5 years of experience, the incidence rate ratio (Exp.B = 1.927) indicates that an increase in the number of nurses within this range significantly increases the incidence of restraints and seclusions. This result suggests that relatively inexperienced nurses are associated with higher rates of restraints and seclusions, The Wald Chi-Square value of approximately 46.27 (*p* < 0.001) indicates a statistically significant relationship between this variable and instances of physical/mechanical restraint and seclusion.

##### Incidence of Physical/Mechanical Restraints and Seclusion and Its Association with Nurses’ Variables for King Fahad University Hospital

Table 11 explores the associations between the occurrence of physical/mechanical restraints and seclusion at the King Fahad University Hospital and nurses’ variables. We employed Negative Binomial Regression and specifically focused on the factors of educational level among nurses with diplomas only and the impact of experience of nurses who had just 1 to 5 years’ worth.

Regarding the total number of nurses in King Fahad University Hospital, the incidence rate ratio (Exp.B = 0.822) suggests that an increase in the total number of nurses tends to be associated with a decrease in the use of restraints and seclusions, although this result is not statistically significant (*p* > 0.05). The Wald Chi-Square value of approximately 3.14 at a significant level of 0.077 indicates a non-statistically significant relationship between the number of nurses and these incidents.

Similarly, regarding the total number of Diploma Nurses in King Fahad University Hospital, the incidence rate ratio (Exp.B = 0.36) implies that increasing the number of diploma-level nurses is associated with a substantial decrease in the incidence of restraints and seclusions. The Wald Chi-Square value of approximately 12.66(*p* < 0.001) indicates a statistically significant association between this variable and the incidence of physical/mechanical restraint and seclusion.

Furthermore, for the total number of nurses with 1 to 5 years’ experience, the incidence rate ratio (Exp.B = 0.141 indicates that an increase in the number of relatively inexperienced nurses significantly reduces the incidence of restraints and seclusions. The Wald Chi-Square value of approximately 26.91 (*p* < 0.001) suggests a statistically significant relationship between this variable and the occurrences of physical/mechanical restraint and seclusion.

These findings underscore the significance of the investigated nursing factors in predicting instances of physical/mechanical restraints and seclusion at King Fahad University Hospital.

## 4. Discussion

This study provides the first prospective data on the incidence and patterns of physical/mechanical restraints and seclusion in Saudi Arabian mental health settings. Over a six-month period, 333 instances of restrictive interventions were recorded among 1388 patients across two facilities. The overall incidence was 0.305 events per patient per month at Eradah Complex and 0.549 events per patient per month at KFUH. These findings establish a baseline for understanding coercive practices in the Saudi context and reveal significant variations associated with patient characteristics, hospital type, and nursing factors. Restraint (physical/mechanical) and seclusion are methods used to prevent harm in mental health settings, and have generated controversial discussions regarding the justification of their use, in many countries, since the beginning of modern psychiatry. This problem of how, how often, and for how long to use restraints remains a challenging issue in psychiatric services and institutions worldwide today [4]. The primary focus of the study was to observe and assess the incidence and use of physical/mechanical restraints and seclusion in two Saudi Arabian psychiatric settings. In our study, we assessed the use of physical/mechanical restraints and seclusion across two psychiatric settings in Saudi Arabia. The observed overall prevalence of these coercive methods was notably high, with 333 instances recorded among 1388 patients admitted during the study period. This rate aligns with findings from previous research, which indicates a persistent reliance on these measures in psychiatric hospitals [4,16]. Such measures are occasionally deemed necessary for managing patients who exhibit severe aggressive behaviours. The estimation of the incidence of these coercive practices was a central focus of this study, providing critical data on the frequency of their use in psychiatric settings. However, it is crucial to note that while restraints and seclusion are used as interventions during acute incidents, they do not constitute standard treatment protocols. The higher incidence of restraint among male patients may be explained by several clinical and behavioural mechanisms. Males with severe mental illness, particularly schizophrenia spectrum disorders, may exhibit more externalising behaviours such as overt aggression and physical agitation, which staff perceive as posing immediate safety risks [17]. Additionally, gender-based differences in help-seeking behaviour and illness presentation may mean that males are admitted during more acute phases of illness, when behavioural dyscontrol is most pronounced. Neurobiological factors, including differences in impulse control and threat perception, may also contribute to this disparity. These findings align with the literature suggesting that males are more likely to experience coercive measures due to both illness presentation and staff responses to male-typical behavioural expressions of distress [18]. This prevalence aligns with other estimates reported in the literature, which demonstrate similar rates of reliance on restraints and seclusion, particularly in managing patients during acute behavioural crises [19,20,21]. These findings highlight the necessity of such measures during extreme incidents, yet their use raises ethical concerns and underscores the importance of balancing safety with patient-centred care. The high prevalence observed in our study emphasizes the ongoing challenge and need for alternative management strategies in psychiatric care settings, aiming to reduce the use of such coercive practices while ensuring the safety of both patients and staff. This reinforces the broader challenge within psychiatric care to identify less restrictive interventions while maintaining safety, as emphasised in previous studies.

When compared with international incidence rates, our findings reveal both similarities and notable differences. The overall incidence of 0.305 events per patient per month at Eradah Complex falls within the range reported in European studies, which vary from 0.1 to 0.8 events per patient per month [4,19]. However, the pattern of intervention type differs markedly. While many European countries have substantially reduced or eliminated mechanical restraint in favour of seclusion or physical holds [16], mechanical restraint remained the predominant intervention in our Saudi settings. This may reflect differences in staff training, available alternatives, and regulatory frameworks. For instance, countries such as the United Kingdom have implemented national reduction programmes with mandatory reporting and oversight [22], whereas Saudi Arabia currently lacks equivalent national policies. The higher use of mechanical restraint in our settings aligns more closely with practices reported in some Asian and Middle Eastern countries [16], suggesting that regional cultural factors and healthcare traditions may influence practice patterns.

Consistent with the international literature [17,23], male patients in our study had a significantly higher incidence of restrictive interventions compared to females. While the precise magnitude of this association could not be reliably estimated due to model instability, the pattern aligns with research suggesting that males may exhibit more externalising behaviours that staff perceive as requiring immediate intervention [18]. This association may also reflect differences in illness presentation, with males more frequently admitted during acute phases of psychosis or agitation.

Diagnosis emerged as a significant factor, with patients diagnosed with substance-related disorders and personality disorders showing higher incidence rates compared to those with schizophrenia spectrum disorders. This finding is consistent with studies reporting that comorbid substance use and personality pathology are associated with increased behavioural dyscontrol and subsequent coercive measures [24,25]. However, given the small sample sizes in some diagnostic subgroups, these estimates should be interpreted cautiously.

In our study, physical/mechanical restraints were notably more likely to be used for durations exceeding 60 min, compared to seclusion. This pattern was observed across both psychiatric settings in Saudi Arabia, where mechanical restraints accounted for a higher proportion of long-duration interventions. Specifically, while seclusion events lasting over 60 min were relatively infrequent, a significant majority of physical/mechanical restraint cases extended beyond this duration. This finding is consistent with the existing literature that suggests mechanical restraints are often applied for longer periods due to the severity or persistence of the behaviours they aim to manage. The extended use of physical restraints underscores the necessity for stringent monitoring and review processes to mitigate potential negative outcomes, including psychological distress and physical harm to patients. This finding aligns with the literature, which suggests that while restraints are intended as a last resort, their application can be prolonged, potentially due to the severity or persistence of the aggressive behaviour [26]. Also, it is important to consider the implications of such extended durations, as they may contribute to adverse outcomes, including physical injuries, psychological distress, and a negative impact on the patient’s therapeutic programme [27]. Knowing that the duration of the restraint can vary, the goal is always to use them for the shortest time necessary to ensure safety. Furthermore, continuous efforts are being made to reduce the use of restraints through preventive strategies [28]. The significant association between duration of the intervention and the type of setting—both in terms of the male/female wards and the admitting department—suggests that these variables influence how long patients are subjected to restraints or seclusion. On the other hand, neither the reason for admission nor the time of restriction influences the duration of the intervention. This could imply that the decision to continue or end an intervention is more dependent on the patient’s response rather than the initial reason of its application or the time of the implementation.

In relation to the time of the restriction, we have found similar proportions of restraint use across different times of the day, and that may suggest that aggressive behaviours necessitating restraints are not confined to any specific time period. This could reflect the unpredictable nature of such behaviours and also underscores the importance of having consistent staffing levels and expertise available at all times to manage incidents safely and effectively. Furthermore, these results may prompt a review of institutional policies and staff training programmes to ensure that restraint use is a last resort, applied uniformly, and not influenced by extraneous factors [22,29].

The finding that the female wards have the lowest proportion of restrictions lasting over 61 min could be indicative of several factors. It may reflect differences in the types of behaviours exhibited by patients in male versus female wards, or the approach staff take in managing these behaviours. Many researchers have shown that the use of seclusion and restraints can be highly variable and influenced by a multitude of factors, including gender dynamics within the ward. Also, it is recognised that women are particularly vulnerable to the distress and trauma associated with these interventions [30]. Furthermore, the significant association between the type of restriction and type of settings among male/female wards suggests that the environment and the patient demographics within a ward can influence the choice of application of restrictive interventions. Along with the duration of the intervention, this is an intriguing aspect of mental health care practices. While the lack of significant association between the type of restriction and the reason for the intervention could suggest that such variables may not influence the decision-making process, it could indicate that the use of such interventions is more individualised and based on immediate clinical judgement rather than being strongly associated with broader reasons, or even locations.

The absence of restraint durations exceeding 60 min in the emergency room (ER) setting is a significant observation. This could be attributed to the fast-paced nature of ERs, where the primary goal is to stabilise patients for immediate care or transfer to specialised units. Restraints in the ER are typically used as a temporary measure to quickly manage acute agitation or violent behaviour, ensuring the safety of the patient and healthcare staff while more definitive treatment or placement is arranged [31]. Additionally, the ER environment may not be conductive to prolonged restraint use due to space constraints and the availability of staff to monitor such situations closely. Accordingly, the emphasis in ER settings is often on rapid de-escalation and the use of short-term emergency interventions rather than extended periods of restraint [32,33].

That violence towards staff and damage to surroundings are the primary reasons for the application of restraints and seclusion is consistent with the literature. These reasons are often cited as the most common triggers for such interventions, as they pose immediate risks to safety and order within the facility. The use of restraints and seclusion is generally viewed as a response to real or potential violence in mental health settings, and these measures are employed to prevent imminent harm to individuals or property when other means of control are ineffective or inappropriate [34]. Other reasons for the use of the restraints and seclusion were also observed, including self-harm and violence towards other patients, among other reasons. The goal is to create a therapeutic environment that minimises the need for such interventions, through patient-specific approaches to the prevention and management of behavioural emergencies.

The distribution of the incidence of the restriction among young patients is a reflection of a demographic that is often represented in psychiatric inpatient settings. Also, the mean age aligns with findings from other coercive measures in younger adult populations, which may be associated with a higher incidence of aggressive behaviours or severe mental health crises in this age group. But the wide age range indicates that though younger adults are more frequently subjected to these interventions, older adults are not exempt. This could be due to various factors, including the presence of age-related psychiatric conditions, cognitive disorders, or presence of pre-existing mental health issues [35]. The predominance of male patients in such interventions is a trend that has been observed in various studies. This gender disparity may be attributed to several factors, including differences in aggressive behaviour patterns, diagnosis prevalence, and even social expectations of male behaviour. Additionally, certain mental health conditions with a higher prevalence among males, such as substance use disorders or antisocial personality disorder, may contribute to situations where restraints or seclusion are necessary [17]. We have observed a high incidence rate among schizophrenia, schizotypal delusional, and other non-mood psychotic disorders. These disorders are often associated with symptoms that can lead to behaviour perceived as disruptive or dangerous, such as hallucinations, delusions, and disorganised thinking. These symptoms can result in agitation, aggression, or self-harm, which may necessitate the use of restraints or seclusion interventions [23,36].

The findings concerning admissions due to the worsening of symptoms, along with those for risk of aggression and suicide, are consistent with the recognised reasons for psychiatric admissions [1,6,23]. The worsening of symptoms can often lead to a deterioration of a patient’s mental state, necessitating acute care to stabilise their condition. This could be due to variety of factors, including the progression of the mental illness, lack of adherence to treatment, or ineffective treatment strategies [24]. Also, the risk of aggression and suicide can pose a significant threat to the safety of the patient, staff, and other patients [25,37]. The findings of the connection between the reason for admission and the age and the gender of the participant may indicate the complex interplay of patient characteristics. The significant correlation with the diagnosis suggests that the underlying psychiatric conditions of patients are closely linked to the reasons why they are admitted to mental health facilities, which in turn may influence the likelihood of experiencing restrictive interventions.

In our study, we observed significant differences in the duration of various restrictive interventions. Specifically, the group subjected to physical/mechanical restraints exhibited a notably higher proportion of extended durations—exceeding 60 min—compared to those in seclusion. This finding underscores the multifactorial nature of mental health care, where various factors including age, gender, diagnosis, type of setting, and reason for admission intricately influence the duration of observation required. Age-related variations suggest that different life stages necessitate varying observation lengths due to distinct clinical risks and presentations. Gender-specific factors may also influence these durations, reflecting the different manifestations and management of mental health symptoms across genders. Notably, certain diagnoses like schizophrenia and mood disorders often require longer observation periods to ensure patient safety, highlighting the complex interplay between clinical needs and treatment approaches. Additionally, the type of setting—whether gender-specific wards or others—impacts the length of observation, potentially due to differences in the levels of supervision and care provided. These observations are critical for understanding and improving the application and management of restrictive interventions in psychiatric settings.

The evidence of a statistically significant decrease in mechanical restraint events within the context of an increase in the observation period is a profound finding that merits through examination. This trend suggests that enhanced monitoring and observation may play a pivotal role in reducing the frequency of mechanical restraints. One explanation for this significant difference is that the individual modifies an aspect of their behaviour in response to their awareness of being observed. In the context of mental health care, the presence of observers could lead to heightened attentiveness and responsiveness from staff. In turn, this potentially leads to more proactive and preventive management of patient’s behaviours and the timely implementation of de-escalation strategies, thereby reducing the necessity for mechanical restraints. Furthermore, the extended observation periods may allow for a more nuanced understanding of each patient’s unique needs and triggers, enabling staff to tailor interventions more effectively. This individualised approach can contribute to a more therapeutic environment, where the emphasis is placed on non-restrictive measures that respect patient autonomy and dignity. The reduction in mechanical restraint events also aligns with contemporary mental health care philosophies that advocate for the minimisation of coercive practices [16,38]. Also, that the increase in the monitoring period correlates with a decrease in seclusion events suggests that the same benefits of increased observation in mental health settings contribute not only to patient well-being, but also to the advancement of care standards. This finding supports the evidence that shows the role of the physical environment plays in the reduction in the use of seclusion.

The decrease in both physical/mechanical restraint and seclusion events alongside an increase in the observation period may indicate several underlying factors that contribute to a more therapeutic environment, ultimately leading to a reduction in the need of such interventions. The increased attention may encourage staff to engage more actively with patients and identify early signs of agitation or distress and apply non-restrictive measures before a situation escalates to the point where restraints or seclusion would be considered. Longer observation periods may allow for more consistent therapeutic engagement with patients and can foster a sense of safety and trust between patients and caregivers, which is crucial in managing challenging behaviours without restoring restrictive practices [39]. This finding may also reflect a broader cultural shift within the observed facilities towards patient-centred care [40].

The significant effect of the risk of aggression, the gender, and the diagnosis on the incidence of restraints and seclusion provides important insight into the factors influencing the use of such interventions. The risk of aggression is a key factor in decisions; this finding was also observed in the literature, which indicates that aggressive incidents are a common precursor to the use of coercive measures [41]. Gender differences in the incidence of events may reflect broader societal, biological, and psychological factors. Males may be more likely to exhibit outwardly aggressive behaviours due to social conditions or higher rates of certain diagnoses, leading to more frequent use of restraints or seclusion. Conversely, females may experience different types of mental health crises that call for alternative interventions [18,42], while certain conditions, such as schizophrenia and personality disorders, are associated with behaviours that can increase the use of restraints and seclusion. Patients with this diagnosis may exhibit severe agitation, psychosis, or a propensity for violence, which can lead to the application of restrictive interventions to manage their symptoms and behaviours. The negative effect of age, and the admitting hospital, suggests that older age groups and certain departments are associated with a lower likelihood of employing these interventions. Older patients may be less likely to be subjected to restraints and seclusion due to a higher prevalence of frailty or medical comorbidities, which can make the use of such interventions riskier. Additionally, older patients might present with different behavioural issues compared to younger patients, which could be managed more effectively with alternative interventions [43].

The physical environment and design of the hospital, staffing levels, and the availability of resources such specialised psychiatric services could also play a role. On the other hand, no effect was observed in relation to the risk of suicide on the incidence of events during the observation period. This could mean that the risk of suicide, while clinically important, may not be a strong predictor of the use of restraints and seclusion compared to other variables; it is possible, though, that the sample could affect the results [44,45]. Results related to nurse/patient ratio suggest that this variable has a different impact on the incidence of the events in both facilities. In view of the Eradah Complex and Mental Health data, this ratio could imply that having fewer nurses available may lead to a higher reliance on restraints and seclusion as a means to manage the situations, possibly due to the reduced ability to provide individualised care and attention. On the other hand, in King Fahad University Hospital, which showed a higher nurse/patient ratio, no significant regression was noted, suggesting no impact on the incidence of the events. This might indicate that other factors, such as staff training, facility policies, or patient population characteristics could be more influential in this setting [44,45,46]. Additionally, the education level of nurses has a varying impact on the incidence of events in both facilities. At King Fahad University Hospital, a higher educational level among nurses correlates with a decrease in these events, while at Eradah Complex and Mental Health, it correlates with an increase in the events. This could suggest that better-educated nurses may be more adapted to employing alternative strategies to manage patient’s behaviour, thus reducing the need for restraints and seclusion. Also, the positive correlation at Eradah Complex and Mental Health could be due to a variety of factors, such as a more challenging patient population, or institutional policies that still favour the use of restraints and seclusion despite the nurse’s higher education levels. With regard to the experience levels at both facilities, Eradah Complex and Mental Health displays the greater experience level along with a positive correlation to incidence of the events; this could imply that the experienced nurses in the facility may be more inclined to use these methods. The increase in the total number of patients correlates with the decrease in the events among both facilities. This could be due to increased peer support among patients, more dynamic and engaging group activities that reduce the need for restraints, or the possibility that, with more patients, there is a greater spread of staff attention and resources, leading to less individual focus on behaviour that might lead to restraints or seclusion [47].

This study has several limitations that should be considered when interpreting the findings. First, data were collected from institutional records, which may be subject to inconsistencies in documentation or reporting practices. Second, the study was conducted in only two hospitals in the Eastern Province, which may limit the generalisability of the findings to other regions of Saudi Arabia. Third, the observational design precludes causal inferences; all associations reported should be interpreted as correlations rather than causal relationships. Fourth, we were unable to adjust for all potential confounders, such as prior history of violence, specific medication regimens, or severity of illness at admission, which may have influenced the use of restrictive interventions. Fifth, the negative binomial regression analysis revealed potential model instability, as evidenced by extremely large coefficients for certain variables, suggesting possible quasi-separation or sparse data in specific subgroups; these estimates should be interpreted with caution. Sixth, the association between observation period and reduced restrictive interventions may be confounded by patient characteristics that influence both length of stay and likelihood of behavioural incidents. Finally, the small number of events in some subgroups necessitated cautious interpretation of certain bivariate analyses.

Despite these limitations, the findings have several implications for mental health practice in Saudi Arabia. The high overall incidence of restrictive interventions suggests an opportunity for reduction through targeted interventions. The strong association between the risk of aggression and subsequent restraint use indicates that improved de-escalation training and early identification of at-risk patients may be beneficial. The variation between facilities suggests that institutional policies and culture play important roles, and that sharing best practices across hospitals could support reduction efforts. The complex relationship between nursing characteristics and restraint use highlights the need for context-specific approaches rather than one-size-fits-all solutions. At a policy level, the absence of national guidelines on restraint reduction in Saudi Arabia represents a gap that could be addressed through adaptation of international frameworks to the local context.

## 5. Conclusions

This first prospective study of restraint and seclusion in Saudi mental health settings reveals a substantial incidence of these practices, with significant variations associated with patient gender, diagnosis, reason for admission, and hospital type. Male patients, those admitted due to risk of aggression, and individuals with substance-related or personality disorders had higher incidence rates. The association between longer observation periods and lower use of restrictive interventions warrants further investigation to understand underlying mechanisms. Notably, the relationship between nursing characteristics and restraint use differed across facilities, suggesting that institutional context moderates these associations. The predominance of mechanical restraint over seclusion distinguishes Saudi practice from some Western countries and highlights the influence of local policies and training. These findings provide a baseline for monitoring future changes and underscore the need for culturally appropriate reduction strategies, improved staff training in de-escalation, and the development of national guidelines. Future research should employ larger, multi-site samples and designs that can better control for confounding to identify effective interventions for reducing coercive practices in this context.

## Figures and Tables

**Table 1:** Descriptive analysis for patient data.

	Psychiatric Hospital (Eradah) (N = 1120)	Teaching Hospital (KFUH) (N = 268)	Overall (N = 1388)
Age			
<19 Y	61 (5.4%)	18 (6.7%)	79 (5.7%)
20–29 Y	332 (29.6%)	97 (36.2%)	429 (30.9%)
30–39 Y	364 (32.5%)	81 (30.2%)	445 (32.1%)
40–49 Y	227 (20.3%)	34 (12.7%)	261 (18.8%)
50–59 Y	104 (9.3%)	24 (9%)	128 (9.2%)
>60 Y	32 (2.9%)	14 (5.2%)	46 (3.3%)
Mean	35.42	34.58	35.26
Median	33	31	33
Range (minimum, maximum)	(18, 81)	(18, 84)	(18, 84)
Std. deviation	10.94	13.109	11.393
Gender			
Male	754 (67.3%)	149 (55.6%)	903 (65.1%)
Female	366 (32.7%)	119 (44.4%)	485 (34.9%)
Diagnosis:			
(F01–F09) Mental disorders due to known physiological conditions	3 (0.3%)	6 (2.2%)	9 (0.6%)
(F10–F19) Mental and behavioural disorders due to psychoactive substance	47 (4.2%)	15 (5.6%)	62 (4.5%)
(F20–F29) Schizophrenia, schizotypal delusional, and other non-mood psychotic disorders	781 (69.7%)	74 (27.6%)	855 (61.6%)
(F30–F39) Mood {affective} disorders	211 (18.8%)	152 (56.7%)	363 (26.2%)
(F40–F48) Anxiety, dissociative, stress-related, somatoform and other nonpsychotic mental disorders	16 (1.4%)	10 (3.7%)	26 (1.9%)
(F50–F59) Behavioural syndromes associated with physiological disturbances and physical factors	2 (0.2%)	0 (0.0%)	2 (0.1%)
(F60–F69) Disorders of adult personality and behaviour	32 (2.9%)	8 (3%)	40 (2.9%)
(F70–F79) Intellectual disabilities	22 (2%)	0 (0%)	22 (1.6%)
(F80–F89) Pervasive and specific developmental disorders	1 (0.1%)	0 (0%)	1 (0.1%)
(F99–F99) Unspecified mental disorder	5 (0.4%)	3 (1.1%)	8 (0.6%)
Reason of Admission:			
Risk of suicide	236 (21.1%)	74 (27.6%)	310 (22.3%)
Risk of aggression	365 (32.6%)	59 (22%)	424 (30.5%)
Worsening of symptoms	432 (38.6%)	122 (45.5%)	554 (39.9%)
Diagnostic uncertainty	28 (2.5%)	0 (0%)	28 (2%)
Risk of moral exposure	6 (0.5%)	0 (0%)	6 (0.4%)
Other	53 (4.7%)	13 (4.9%)	66 (4.8%)
Type of setting			
Male ward	449 (40.1%)	82 (30.6%)	531 (38.3%)
Female ward	187 (16.7%)	61 (22.8%)	248 (17.9%)
ER	484 (43.2%)	125 (46.6%)	609 (43.9%)

**Table 2:** Descriptive analysis for restrictive data.

Restrictive Data	Overall (N = 333)	Mechanical/physical Restraint (N = 238)	Seclusion (N = 95)
Duration of restrictions:			
0–30 min	120 (36%)	89 (37.4%)	31 (32.6%)
31–60 min	158 (47.4%)	100 (42%)	58 (61.1%)
61–90 min	8 (2.4%)	7 (2.9%)	1 (1.1%)
91–120 min	34 (10.2%)	30 (12.6%)	4 (4.2%)
121+ min	13 (3.9%)	12 (5%)	1 (1.1%)
Time of restrictions:			
Morning (4 a.m. to 12 p.m.)	92 (27.6%)	66 (27.7%)	26 (27.4%)
Afternoon (12 p.m. to 5 p.m.)	89 (26.7%)	69 (29%)	20 (21.1%)
Evening (5 p.m. to 9 p.m.)	89 (26.7%)	64 (26.9%)	25 (26.3%)
Night (9 p.m. to 4 a.m.)	63 (18.9%)	39 (16.4%)	24 (25.3%)
Type of hospital:			
Psychiatric	275 (82.6%)	199 (83.6%)	76 (80%)
Teaching	58 (17.4%)	39 (16.4%)	19 (20%)
Type of setting:			
Male	209 (62.8%)	188 (79%)	21 (22.1%)
Female	116 (34.8%)	43 (18.1%)	73 (76.8%)
ER	8 (2.4%)	7 (2.9%)	1 (1.1%)
Department:			
Psychiatric Male 1	31 (9.3%)	25 (10.5%)	6 (6.3%)
Psychiatric Male 2	94 (28.2%)	89 (37.4%)	5 (5.3%)
Psychiatric Male 3	43 (12.9%)	41 (17.2%)	2 (2.1%)
Psychiatric Female 1	50 (15%)	19 (8%)	31 (32.6%)
Psychiatric Female 2	52 (15.6%)	20 (8.4%)	32 (33.7%)
Psychiatric ER	5 (1.5%)	5 (2.1%)	0 (0%)
Teaching Male	41 (12.3%)	33 (13.9%)	8 (8.4%)
Teaching Female	14 (4.2%)	4 (1.7%)	10 (10.5%)
Teaching ER	3 (0.9%)	2 (0.8%)	1 (1.1%)
Violence to self			
Yes	84 (25.2%)	61 (25.6%)	23 (24.2%)
No	249 (74.8%)	177 (74.4%)	72 (75.8%)
Violence to staff			
Yes	111 (33.3%)	85 (35.7%)	26 (27.4%)
No	222 (66.7%)	153 (64.3%)	69 (72.6%)
Violence to patients			
Yes	82 (24.6%)	57 (23.9%)	25 (26.3%)
No	251 (75.4%)	181 (76.1%)	70 (73.7%)
Damage to environment			
Yes	93 (27.9%)	69 (29%)	24 (25.3%)
No	240 (72.1%)	169 (71%)	71 (74.7%)
Other			
Yes	35 (10.5%)	23 (9.7%)	12 (12.6%)
No	298 (89.5%)	215 (90.3%)	83 (87.4%)

**Table 3:** Descriptive analysis for daily data of nursing characteristics at Eradah Complex and Mental Health and KFUH.

		ERADA Hospital	KFUH
Variables		Median (Min.–Max.)	Median (Min.–Max.)
Nurses/patient ratio		0.25 (0.19–0.32)	1.1 (0.67–1.89)
Educational level of nurses in the unit	Number of diploma nurses	28 (19–40)	5 (3–6)
	Number of bachelor nurses	9 (2–15)	12 (7–17)
	Number of nurses less than 1 y experience	4 (0–8)	1 (0–2)
Experience of nurses	Number of nurses 1–5 y experience	9 (4–15)	1 (0–2)
	Number of nurses 6–10 y experience	14 (8–22)	6 (1–9)
	Number of nurses more than 10 y experience	11 (5–19)	9 (5–13)

**Table 4:** Association between type of restrictions, type of hospital, and type of setting, and the duration of restrictions.

	Restrictions Data		Duration of Restrictions		*p*-Value
		0–30 min	31–60 min	61+ min	
Type of restrictions	Mechanical/physical	89 (74.2%)	100 (63.3%)	49 (89.1%)	<0.001 *
	Seclusion	31 (25.8%)	58 (36.7%)	6 (10.9%)	
Type of hospital	Psychiatric hospital	99 (82.5%)	142 (89.9%)	34 (61.8%)	<0.001 *
	Teaching hospital	21 (17.5%)	16 (10.1%)	21 (38.2%)	
Type of setting	Male ward	77 (64.2%)	87 (55.1%)	45 (81.8%)	<0.001 *
	Female ward	37 (30.8%)	69 (43.7%)	10 (18.2%)	
	ER	6 (5%)	2 (1.3%)	0 (0%)	

* Due to expected cell counts of less than 5 in one cell, the *p*-value reported is from Fisher’s Exact Test.

**Table 5:** Association between the type of restrictions and the type of setting.

	Restrictions Data	Type of Restrictions		*p*-Value
		Physical/Mechanical Restraint	Seclusion	
Type of setting	Male ward	188 (79%)	21 (22.1%)	<0.001 *
	Female ward	43 (18.1%)	73 (76.8%)	
	ER	7 (2.9%)	1 (1.1%)	

* Due to expected cell counts of less than 5 in one cell, the *p*-value reported is from Fisher’s Exact Test.

**Table 6:** Association between the patients’ characteristics and the reason for admission.

			Reason for Admission (N = 1231)			* *p*-Value
			Risk of Suicide	Risk of Aggression	Worsening of Symptoms	
Age	Mean		33.46	34.96	36.49	<0.001
	Median		31	32	34	
	Std. deviation		11.77	11.01	11.22	
	Range		(18–67)	(18–69)	(18–84)	
Gender	Male	N (%)	174 (56.1%)	330 (77.8%)	352 (63.5%)	<0.001
	Female	N (%)	136 (43.9%)	94 (22.2%)	202 (36.5%)	
Diagnosis	(F10–F19) Mental and behavioural disorders due to psychoactive substance	N (%)	19 (6.6%)	24 (5.8%)	15 (2.8%)	<0.001
	(F20–F29) Schizophrenia, schizotypal delusions, and other non-mood psychotic disorders	N (%)	128 (44.6%)	291 (70.6%)	372 (69.9%)	
	(F30–F39) Mood {affective} disorders	N (%)	120 (41.8%)	90 (21.8%)	134 (25.2%)	
	(F60–F69) Disorders of adult personality and behaviour	N (%)	20 (7%)	7 (1.7%)	11 (2.1%)	
Setting	Male ward	N (%)	101 (32.6%)	214 (50.5%)	191 (34.5%)	<0.001
	Female ward	N (%)	65 (21%)	52 (12.3%)	112 (20.2%)	
	ER	N (%)	144 (46.5%)	158 (37.3%)	251 (45.3%)	
Hospital	Psychiatric hospital	N (%)	236 (76.1%)	365 (86.1%)	432 (78%)	<0.001
	Teaching hospital	N (%)	74 (23.9%)	59 (13.9%)	122 (22%)	

* *p*-value for age derived from Kruskal–Wallis test.

**Table 7:** Association between the patients’ characteristics and duration of observation.

		Duration of Observation (Days)				*p*-Value
		<30	30–60	61–90	>90	
Age	Mean	34.91	35.82	33.51	38.68	=0.068
	Median	32	34	31	37	
	Std. deviation	11.44	10.5	9.76	12.2	
	Range	(18, 84)	(19, 67)	(18, 63)	(19, 65)	
Gender	Male	689 (64.3%)	114 (77.6%)	36 (65.5%)	64 (56.1%)	=0.16
	Female	383 (35.7%)	33 (22.4%)	19 (34.5%)	50 (43.9%)	
Diagnosis	(F10–F19) Mental and behavioural disorders due to psychoactive substance	52 (5%)	7 (4.9%)	2 (3.8%)	1 (0.9%)	<0.008
	(F20–F29) Schizophrenia, schizotypal delusional, and other non-mood psychotic disorders	630 (60.4%)	106 (73.6%)	40 (75.5%)	79 (73.1%)	
	(F30–F39) Mood {affective} disorders	307 (29.4%)	26 (18.1%)	9 (17%)	21 (19.4%)	
	(F40–F48) Anxiety, dissociative, stress-related, somatoform and other nonpsychotic mental disorders	22 (2.1%)	2 (1.4%)	0 (0%)	2 (1.9%)	
	(F50–F59) Behavioural syndromes associated with physiological disturbances and physical factors	2 (0.2%)	0 (0%)	0 (0%)	0 (0%)	
	(F60–F69) Disorders of adult personality and behaviour	30 (2.9%)	3 (2.1%)	2 (3.8%)	5 (4.6%)	
Setting	Male ward	317 (29.6%)	114 (77.6%)	36 (65.5%)	64 (56.1%)	<0.001
	Female ward	146 (13.6%)	33 (22.4%)	19 (34.5%)	50 (43.9%)	
	ER	609 (56.8%)	0 (0%)	0 (0%)	0 (0%)	
Hospital	Psychiatric Hospital	839 (78.3%)	127 (86.4%)	46 (83.6%)	108 (94.7%)	=0.098
	Teaching Hospital	233 (21.7%)	20 (13.6%)	9 (16.4%)	6 (5.3%)	
Reason of admission	Risk of suicide	258 (26.1%)	28 (20%)	6 (11.5%)	18 (17%)	<0.001
	Risk of aggression	308 (31.1%)	55 (39.3%)	25 (48.1%)	36 (34%)	
	Worsening of symptoms	424 (42.8%)	57 (40.7%)	21 (40.4%)	52 (49.1%)	

**Table 8:** Incidence of physical/mechanical restraints, incidence of seclusions in Eradah Complex and Mental Health and KFUH.

Parameter	* B	* Std. Error	* Wald Chi-Square (χ^2^)	* Sig.	* Exp(B)	* Exp(B) × 30.5
Eradah						
Number of mechanical restraints	−4.95	0.087	319.16	0.00	0.007	0.2135
Number of seclusions	−5.99	0.127	2217.92	0.00	0.003	0.0915
Number of restraints and seclusion	−4.617	0.0784	3466.82	0.00	0.010	0.3050
KFUH						
Number of mechanical restraints	−4.42	0.19	537.76	0.000	0.012	0.3660
Number of seclusions	−5.24	0.256	418.84	0.000	0.005	0.1525
Number of restraints and seclusion	−4.019	0.165	595.05	0.000	0.018	0.5490

* B: Logarithm of the expected count. Std. Error: Standard error of the coefficient. Wald Chi-Square (χ^2^): Statistical test assessing the contribution of the variable to the model. Sig.: *p*-value indicating statistical significance (<0.05 is significant). Exp(B): Incidence Rate Ratio (IRR) representing the estimated daily rate of events per patient. Exp(B) × 30.5: Estimated monthly rate of events per patient (daily IRR multiplied by 30.5, the average number of days per month).

**Table 9:** Patients’ factors associated with the incidence of physical/mechanical restraints and incidence of seclusions in Eradah Complex and Mental Health and KFUH.

Factor	Categorisation	* Percent	* B	* Std. Error	* Wald Chi-Square (χ^2^)	* Sig	* Exp(B)
	Ref. group = Females, 33.2%						
Gender	Male	66.8%	25.961	0.4103	4004.52	0.000	1.8 × 10^10^
	Ref. group = Disorders of adult personality and behaviour, 3.1%						
Diagnosis	Mental and behavioural disorders due to psychoactive substance	4.7%	2.383	0.7137	11.145	0.001	10.83
	Schizophrenia, schizotypal delusional, and other non-mood psychotic disorders	64.3%	1.593	0.6629	5.777	0.016	4.92
	Mood {affective} disorders	27.9%	1.746	0.6717	6.759	0.009	5.733
	Ref. group = Worsening of symptoms, 43.2%						
Reason for Admission	Risk of suicide	23.3%	0.317	0.2190	2.090	0.148	1.372
	Risk of aggression	33.5%	0.468	0.1775	6.968	0.008	1.598
	Ref. group = setting 3, 42.5%						
Type of settings	Male	39.5%	−0.546	0.3985	1.880	0.170	0.579
	Female	18.0%	25.755	.	.	.	15 × 10^10^
Age			−0.043	0.008	29.897	0.00	0.958
	Ref. group = KFUH, 19.3%						
Hospital	Eradah	80.7%	−0.444	0.207	4.589	0.032	0.641

* Percent: Proportion of participants in each category. B: Logarithm of the odds ratio compared to the reference group. Exp(B): Odds ratio indicating the likelihood of the outcome. Sig: *p*-value showing statistical significance (<0.05 is significant). Wald Chi-Square (χ^2^): Contribution of the variable to the model. Std. Error: Variability in the coefficient estimate.

**Table 10:** Associations with physical/mechanical restraint and seclusion for Hospital Eradah Complex and Mental Health.

Parameter	B	Std. Error	Wald Chi-Square (χ^2^)	Sig	Exp(B)
Total nurses	−0.476	0.0902	27.80	<0.001	0.621
Total diploma-level nurses	0.302	0.0900	11.25	0.001	1.352
Total 1–5 y experience	0.656	0.0964	46.27	<0.001	1.927

**Table 11:** Associations of physical/mechanical restraint and seclusion with nurses’ factors for King Fahad University Hospital.

Parameter	B	Std. Error	Wald Chi-Square (χ^2^)	Sig	Exp(B)
Total. Nurses	−0.196	0.1105	3.14	0.077	0.822
Total diploma-level nurses	−1.036	0.2911	12.66	<0.001	0.36
Total 1–5 y experience	−1.960	0.3778	26.914	<0.001	0.141

## Data Availability

The data are not publicly available due to Institutional Review Board (IRB) regulations, including data security requirements and participant confidentiality. Access to the data is therefore restricted, and further inquiries may be directed to the corresponding author, subject to ethical approval.
